# Strengthening the Aging Brain: Functional Connectivity Changes After a Language-Based Cognitive Program

**DOI:** 10.3390/brainsci15111139

**Published:** 2025-10-24

**Authors:** Anne-Sophie Beaumier, Ana Paula Bastos, Bárbara Malcorra, Bárbara Rusch da Rocha, Vanessa Bisol, Fernanda Souza Espinosa Borges, Erica dos Santos Rodrigues, Maria Teresa Carthery-Goulart, Lucas Porcello Schilling, Karine Marcotte, Lilian Cristine Hübner

**Affiliations:** 1 Département de Psychologie, Faculté des Arts et Sciences, Université de Montréal, Montréal, QC H2V 2S9, Canada; 2 Research Center of Centre Intégré Universitaire de Santé et de Services Sociaux du Nord-de-l’Île-de-Montréal, Montréal, QC H4J 1C5, Canada; 3 Graduate Course in Linguistics, School of Humanities, Pontifical Catholic University of Rio Grande do Sul (PUCRS), Porto Alegre 90619-900, RS, Brazil; 4 Psychology Department, School of Health and Life Sciences, Pontifical Catholic University of Rio Grande do Sul (PUCRS), Porto Alegre 90619-900, RS, Brazil; 5 Department of Letters and Performing Arts, Center for Theology and Human Sciences, Pontifical Catholic University of Rio de Janeiro (PUC-Rio), Rio de Janeiro 22451-900, RJ, Brazil; 6 National Council for Scientific and Technological Development (CNPq), Brasília 70730-500, DF, Brazil; 7 Center for Mathematics, Computing, and Cognition, Federal University of ABC (UFABC), Santo André 09280-560, SP, Brazil; 8 Human Communication, Learning, and Development Unit, Faculty of Education, The University of Hong Kong, Hong Kong, China; 9 Cognitive and Behavioral Neurology Unit, Department of Neurology, Hospital das Clínicas, University of São Paulo, São Paulo 05508-220, SP, Brazil; 10 Brain Institute of Rio Grande do Sul, Pontifical Catholic University of Rio Grande do Sul (PUCRS), Porto Alegre 90619-900, RS, Brazil; 11 Graduate Course of Gerontology and Geriatrics, School of Medicine, Pontifical Catholic University of Rio Grande do Sul (PUCRS), Porto Alegre 90619-900, RS, Brazil; 12 Institute of Geriatrics and Gerontology (IGG), Pontifical Catholic University of Rio Grande do Sul (PUCRS), Porto Alegre 90619-900, RS, Brazil

**Keywords:** linguistic intervention, aging, functional connectivity, cognitive training, language, language processing, cognitive reserve

## Abstract

**Background/Objectives:** Accumulating evidence suggests that cognitive training can induce functional reorganization of intrinsic connectivity networks involved in higher-order cognitive processes. However, few interventions have specifically targeted language, an essential domain tightly interwoven with memory, attention, and executive functions. Given their foundational role in communication, reasoning, and knowledge acquisition, enhancing language-related abilities may yield widespread cognitive benefits. This study investigated the neural impact of a new structured, language-based cognitive training program on neurotypical older adults. **Methods:** Twenty Brazilian Portuguese-speaking women (aged 63–77 years; schooling 9–20 years; low-to-medium socioeconomic status) participated in linguistic activities designed to engage language and general cognitive processing. Behavioral testing and resting-state functional Magnetic Resonance Imaging (fMRI) were conducted before and after the intervention. **Results:** Functional connectivity analyses revealed significant post-intervention increases in connectivity within the frontoparietal network, critical for language processing, and the ventral attentional network, associated with attentional control. **Conclusions**: The observed neural enhancements indicate substantial plasticity in cognitive networks among older adults, highlighting the effectiveness of linguistic interventions in modulating critical cognitive functions. These findings provide a foundation for future research on targeted cognitive interventions to promote healthy aging and sustain cognitive vitality.

## 1. Introduction

Cognitive changes are well-documented aspects of both neurotypical and pathological aging. Changes in several cognitive domains are expected to occur with advancing age, including reductions in working memory capacity, processing speed, and attentional control. For example, older adults often exhibit slower reaction times and increased interference in tasks that require attentional control, such as the Stroop task [1,2]. Executive functioning is particularly relevant because its decline is a significant predictor of Alzheimer’s disease (AD) [3,4] together with episodic memory [5]. Preserving cognitive functions may help delay or mitigate functional decline or, at the very least, contribute to maintaining or improving cognition.

Over the past two decades, there has been growing interest in cognitive training interventions aimed at improving cognitive function in older adults. These interventions vary in modality (e.g., online vs. face-to-face), duration, frequency, and target domains (domain-specific vs. general cognition). Studies have demonstrated that cognitive training can enhance cognitive performance in older adults with cognitive impairment (e.g., [6,7,8,9,10]) and without (e.g., [11,12,13,14,15,16]). For instance, Corbett et al. [12] reported significant benefits from online cognitive training interventions targeting reasoning and general cognitive abilities, noting improvements in self-reported instrumental activities of daily living (IADL) among adults aged over 60 years. This study further indicated considerable positive effects on reasoning and verbal learning, as well as moderate gains in spatial working memory, compared with control participants. Similarly, Borella et al. [11] reported positive outcomes specifically related to working-memory-focused cognitive training programs. Moreover, cognitive training interventions have shown lasting benefits. Long-term evidence from the Advanced Cognitive Training for Independent and Vital Elderly (ACTIVE) study indicated that cognitive improvements persisted for up to 10 years post-intervention, notably in areas such as IADL, reasoning, and processing speed [14]. Earlier findings from the same program similarly showed sustained benefits in IADL and cognitive performance lasting up to 5 years [15]. From a neurophysiological perspective, numerous studies have shown that cognitive training interventions can affect brain activity. Consistent neural changes have been identified primarily within the frontoparietal network (FPN), including alterations in the medial frontal gyrus, precentral gyrus, inferior frontal gyrus, superior parietal cortex, supramarginal gyrus, and superior temporal gyrus [17,18,19,20].

Most cognitive training programs are delivered either online or face-to-face, primarily targeting specific cognitive domains such as executive functions [21] or episodic memory [22]. However, interventions that solely adopt linguistic stimuli are comparatively rare [23,24]. Moreover, studies examining the impact of language-based training on broader cognitive functions are scarce [25]. However, language processing fundamentally underpins cognitive performance. For instance, reading and writing habits in neurotypical older adults significantly mitigate age-related cognitive decline [26]. Additionally, semantic memory, as reflected by speech connectivity measures, is closely linked to cognitive functioning in Alzheimer’s disease [26]. Linguistically oriented interventions may be particularly beneficial for populations with lower socioeconomic status (SES), whose educational attainment and literacy levels are typically lower.

Despite the well-established relationship between language and other cognitive functions, interventions that specifically utilize language-based tasks remain limited. Language processing heavily depends on cognitive functions, such as executive control, which supports comprehension by resolving ambiguities in sentences and enabling efficient word retrieval by inhibiting competing lexical alternatives [27]. Similarly, working memory substantially contributes to language comprehension by allowing listeners to maintain earlier parts of sentences while processing subsequent segments [28]. Executive functions have also been demonstrated to influence everyday language use in older adults; higher executive abilities correlate positively with more analytical linguistic expressions such as using longer words and more frequent numerical references [29]. Furthermore, deficits in semantic short-term memory are associated with impaired word production and sentence comprehension, which reflects increased lexical competition due to inhibitory dysfunction [30]. Attention, together with other general cognitive skills, is intrinsically related to language processing at both the comprehension and production levels, providing insights into the relationship between language, cognition, and brain-related circuitry [31]. Understanding these cognitive–language interdependencies emphasizes the potential effectiveness of language-based cognitive training interventions in promoting cognitive health.

To better understand how cognitive training may shape the neural mechanisms supporting cognitive–linguistic interactions in aging, resting-state functional magnetic resonance imaging (fMRI) offers a powerful approach for examining the intrinsic organization and plasticity of large-scale brain networks that underlie cognitive and language functions. By assessing synchronized activity among spatially distinct brain regions, resting-state fMRI enables the identification of intrinsic connectivity networks that are fundamental to cognitive processes including language. Among these, the default mode network (DMN), frontoparietal network (FPN), dorsal attention network (DAN), ventral attention network (VAN), and salience network (SN) are particularly relevant because of their involvement in language and cognitive functions. The DMN primarily supports internally directed cognitive activities, such as autobiographical memory, planning, social cognition, and executive processes, such as working memory and cognitive flexibility. It is active at rest but deactivates during goal-directed tasks, with reduced connectivity linked to cognitive decline and memory impairment in aging and Alzheimer’s disease [32]. In contrast, the FPN, also commonly referred to as the central executive network (CEN) or executive control network (ECN), serves as a cognitive control hub crucial for executive functions, including cognitive flexibility, working memory, and semantic control [33,34]. The FPN dynamically interacts with the DMN and attention networks, facilitating shifts between internal and external cognitive states and modulating distractions [35,36,37].

Attention is regulated primarily by two complementary networks. The DAN directs attention toward relevant stimuli and is essential for working memory and memory encoding, influencing tasks such as reading by modulating attention to visual and spatial cues [38,39]. Meanwhile, VAN detects and reorients attention to unexpected stimuli, contributing to cognitive flexibility and reading fluency [40,41]. Central to orchestrating these interactions is the SN, which monitors and filters relevant stimuli, and facilitates cognitive flexibility by alternating between internally focused processes (DMN-driven) and externally directed tasks (FPN/DAN-driven). Key regions of the SN, including the anterior insula and anterior cingulate cortex, have significant implications for language functions, such as articulation, word retrieval, and phonological discrimination [42,43,44]. Moreover, SN connectivity patterns have been linked to age-related decline in cognitive functions, particularly executive control, working memory updating, and attention regulation [45].

Accumulating evidence suggests that cognitive training can induce functional reorganization in intrinsic connectivity networks implicated in higher-order cognitive processes. For instance, Cao et al. [46] reported maintained or increased resting-state functional connectivity within the DMN, FPN, and SN in healthy older adults following a one-year cognitive training intervention. Similarly, Chapman et al. [47] demonstrated increased functional connectivity within the DMN and PFN after a shorter (12-week) program, with connectivity increases correlating with cognitive gain. Consistent with these findings, De Marco et al. [48] also reported strengthened connectivity within the DMN after just one month of computerized cognitive training focused specifically on this network.

However, not all studies reported uniform findings across networks. For instance, Hardcastle et al. [49] identified significant increases solely within the FPN following a multi-domain cognitive intervention, attributing the absence of detectable DMN and DAN changes to potentially high cognitive reserves in their highly educated sample, suggesting that a longer duration might be necessary to reveal changes in these networks. Additionally, Strenziok et al. [50] proposed that DAN connectivity changes specifically underpin the mechanisms of far transfer, and their results indicated reduced connectivity between the superior parietal cortex (part of the DAN) and inferior temporal regions exclusively when far transfer occurred [51]. Furthermore, only a few studies have specifically examined the effects of cognitive training on VAN connectivity. Nevertheless, Hampstead et al. [52] reported an enhanced VAN connectivity around the temporoparietal junction following an explicit training program. Taken together, these findings highlight the capacity of cognitive training to reshape the architecture of large-scale brain networks and underscore the importance of the possibility that language-centered interventions might similarly modulate these networks, and consequently, cognitive functions.

Finally, further evidence on the impact of language-based training, cognition, and brain circuitry has been provided by studies on bilingualism, second language training, and functional illiteracy. Research on bilingualism has demonstrated enhanced executive function in multilingual individuals, including improved monitoring, inhibition, attention, and delayed cognitive decline [53,54,55]. Furthermore, a few studies have explored the direct impact of second-language-based interventions on cognitive function. For instance, a four-month second-language learning intervention in older adults resulted in improved global cognition and increased connectivity within language and executive networks, suggesting a promising avenue for cognitive interventions focusing on language [56]. Complementing these findings, Mohammadi et al. [57] reported that literacy training in functional illiterates reduced hyperconnectivity within the left FPN, suggesting a compensatory mechanism by which executive resources initially support cognitive deficits [56]. These findings collectively underscore the interconnected roles of language, cognitive functions, and their underlying neural networks, suggesting that targeted cognitive interventions leveraging linguistic training may effectively enhance cognitive function in older adults.

Building upon prior evidence, the present study aimed to determine whether language-based cognitive intervention can induce changes in resting-state functional connectivity in healthy older adults. Specifically, we examined whether a two-month linguistic training program modulated connectivity within large-scale brain networks associated with language processing and cognitive functions. At the behavioral level, we hypothesized improvements in episodic memory [25]. At the neurobiological level, we predicted increased functional connectivity in the DMN [46,47,48] and FPN [47,49,57], both of which have been implicated in cognitive training-related plasticity. We also anticipate subtle changes in the SN [46] and either preserved or reduced connectivity in the DAN, consistent with findings suggesting compensatory reallocation [49,50,51]. Although the ventral attention network (VAN) has received limited attention in this context, we explored the possibility of enhanced connectivity based on previous work [52]. By elucidating these neurofunctional adaptations, our study sought to establish the efficacy of language-based cognitive training as a targeted approach to support brain health in aging.

## 2. Materials and Methods

### 2.1. Participants

Twenty neurologically healthy individuals were recruited from a metropolitan area in southern Brazil. Three participants were excluded from fMRI data analysis due to excessive head motion or global signal fluctuations during fMRI acquisition. Only women were included in this study due to difficulties recruiting men. The demographic and clinical characteristics of the participants are summarized in Table 1. Their ages ranged from 63 to 77 years (mean = 69.3, SD = 4.7), and their years of schooling ranged from 9 to 20 years (mean = 14.85, SD = 3.3). To be included, participants were required to be native Brazilian Portuguese speakers, right-handed, literate, and eligible for fMRI. Exclusion criteria included a history of major psychiatric disorders, learning disabilities, severe or uncorrected self-reported perceptual deficits, and any additional neurological diagnoses. Handedness was assessed using the Edinburgh Handedness Inventory [58]. All participants provided written informed consent before enrollment. The study was conducted in accordance with the Declaration of Helsinki and approved by the Ethics Committee of Pontifícia Universidade Católica do Rio Grande do Sul (PUCRS)-protocol number 53696221.4.1001.5336, approved on 18 January 2022) 

### 2.2. Materials and Procedures

Participants underwent a complete assessment before and after cognitive training, which included fMRI as well as language, cognitive, and neuropsychological assessments.

#### 2.2.1. Sociodemographic, Emotional, Functional and Cognitive Assessment

Questionnaires were administered to gather information about the participants’ language profiles, socialization habits, physical training habits, and general health. Socioeconomic status (SES) was determined using Critério Brasil 2024 [59]. SES scoring is based on household characteristics, the schooling level of the household head, and the availability of consumer goods and amenities. The scores are categorized into six socioeconomic groups, from highest to lowest: A (45–100 points), B1 (38–44 points), B2 (29–37 points), C1 (23–28 points), C2 (17–22 points), and DE (0–16 points). Reading and writing habits (RWH) were assessed using a questionnaire developed in Brazil [60]. This instrument includes 30 questions assessing the frequency of engaging in various reading activities (e.g., magazines, newspapers, books, social media) and writing tasks (e.g., notes, text messages, literary and non-literary texts) as well as the participants’ history of literacy development. Scores range from 0 to 100, with lower scores indicating more frequent engagement in reading and writing.

Participants were assessed for depressive symptoms using the Brazilian version of the Geriatric Depression Scale (GDS) [61] and for functional impairments indicative of cognitive decline or dementia using the Functional Activities Questionnaire (FAQ) [62]. Anxiety symptoms were evaluated using the Geriatric Anxiety Inventory (GAI) [63]. Furthermore, participants underwent a comprehensive neuropsychological assessment that included the Brazilian version of Addenbrooke’s Cognitive Examination–Revised (ACE-R, [64,65], the Mini-Mental State Examination (MMSE) [66], the Rey Auditory Verbal Learning Test (RAVLT) [67], the Digit Span of the WAIS-III [68], the Victoria Stroop Test [69], the Camel and Cactus Test (CCT) [70], the the standardized Brazilian version [71] of the Colors Trails Test (CTT), phonemic verbal fluency (“p”), semantic verbal fluency (animals) and a naming task (subtest of the Battery for Language Assessment in Aging (BALE)) [72].

#### 2.2.2. MRI Protocol

All MRI data were acquired using a Siemens Magnetom Vida 3.0T HDxt scanner. For structural imaging, a high-resolution three-dimensional (3D) T1-weighted scan was obtained using a magnetization-prepared rapid gradient echo (MP-RAGE) sequence. The imaging parameters were as follows: the phase encoding direction was anterior–posterior, the repetition time (TR) was 2300 ms, the echo time (TE) was 2.98 ms, and the inversion time (TI) was 900 ms. The field of view (FOV) was 256 × 256 mm, with a voxel size of 1 × 1 × 1 mm^3^ and a slice thickness of 1 mm. A multiband acceleration factor of three was used with a matrix size of 256 × 256 mm, yielding a total of 176 slices. The data were collected using a 16-channel skull coil.

For functional imaging, a gradient-recalled echo planar imaging (GRE-EPI) sequence was used to acquire a total of 462 scans. The imaging parameters included anterior–posterior phase-encoding direction and interleaved slice acquisition. The TR was 1280 ms, and the TE was 35 ms. The FOV was 212 × 212 × 212 mm, with a voxel size of 2.4 × 2.4 × 2.4 mm^3^ and a slice thickness of 2.4 mm. A multiband acceleration factor of four was applied, with a matrix size of 256 × 256 mm and 60 slices acquired per volume. Functional data were collected by using a 16-channel skull coil.

#### 2.2.3. Cognitive Training

The linguistic–cognitive training intervention lasted 16 weeks, consisting of one weekly 90 min in-person group session and four individual sets of activities for home-practice. Each session included supplementary pen-and-paper activities distributed to participants, who were instructed to complete them individually at home between meetings. These home activities consisted of five tasks per day, conducted four days per week (excluding weekends and session days), amounting to approximately 30–40 min of daily individual practice.

Activities encompassed reading and comprehension of short texts from different genres, tasks requiring inference and analysis of text structure, and logical reasoning exercises based on short text analyses. Participants also engaged in short writing activities, including personal narratives, news summaries, notes, messages, and collaborative story writing. Vocabulary-focused tasks emphasized word generation, exploration of word meanings, and syntactic exercises such as sentence reordering and the analysis of active versus passive constructions. Importantly, the activities were not organized in a linear progression of difficulty; instead, they integrated analyses at word, sentence, and text levels to sustain participants’ engagement.

The group sessions emphasized dynamic interactions, combining individual and collaborative activities with collective feedback and corrections. Sessions utilized pre-prepared materials presented via a projector, incorporating engaging formats such as board games, music-based language activities, oral narrative exercises, and speech tasks. The session structure was as follows: (1) approximately 5 min dedicated to welcoming participants and conducting a warm-up; (2) approximately 15–20 min for group correction and discussion of home activities; (3) 50–60 min allocated to core language-based cognitive training tasks performed collaboratively; and (4) 5 min to express appreciation to participants, summarizing session achievements, and distributing materials and instructions for the upcoming week’s individual tasks.

### 2.3. Analysis

#### 2.3.1. Statistical Analysis of the Behavioral Data

Descriptive statistics (mean, standard deviation, minimum, and maximum) were computed for the test variables at the baseline. To examine the changes from pre-to post-intervention, paired comparisons were conducted for each cognitive and self-report measure. Shapiro–Wilk tests were conducted to assess the normality of the difference scores. Due to violations of normality in several variables (*p* < 0.05), the non-parametric Wilcoxon Signed-Rank Test was employed. To correct for multiple comparisons, *p*-values were adjusted using the Benjamini (BH) procedure [73]. A significance threshold of *p* < 0.05 (adjusted) was adopted for identifying statistically significant changes. The data were processed using R (version 4.3.1) [74] in the R Studio environment [75], version 2025.05.0+496.pro5.

#### 2.3.2. Functional Connectivity Preprocessing

MRI data pre-processing and statistical analyses were conducted using the CONN toolbox (RRID:SCR_009550, version 22.v2407) [76] and SPM12 (Wellcome Department of Imaging Neuroscience, London, UK) [77] implemented in MATLAB R2024a (MathWorks, Natick, MA, USA) [78]. Preprocessing followed CONN’s standardized pipeline of the CONN [79], which included realignment with susceptibility distortion correction [80], slice-timing correction [81,82] outlier detection [83], segmentation, normalization to MNI space, and spatial smoothing with an 8 mm FWHM Gaussian kernel.

Functional images were realigned to the first scan of the first session using 6-parameter rigid-body transformation [84] and B-spline interpolation to correct motion and susceptibility interactions. Temporal misalignment due to interleaved slice acquisition was corrected using sinc interpolation to a mid-acquisition reference time. Prior to connectivity analysis, data quality was assessed for all participants. Three individuals were excluded because of excessive head motion or global signal fluctuations during fMRI acquisition, which rendered their data unsuitable for reliable connectivity analysis. Specifically, two participants exceeded motion thresholds (framewise displacement > 0.5 mm), and one participant exhibited excessive global signal changes (>3 SD), as identified by Artifact Detection Tools [85].

Anatomical and functional images were segmented into gray matter, white matter, and CSF and normalized to a 2 mm isotropic MNI space using SPM’s unified segmentation and normalization algorithm [86,87] with the IXI-549 tissue probability maps [88]. Denoising included the regression of five white matter and five principal components from white matter and CSF (CompCor) [89,90] and their first-order derivatives, outlier scans, session effects, and linear trends. CompCor components were extracted from the eroded tissue masks and orthogonalized with respect to the average BOLD signal, motion, and outliers. Following the nuisance regression, a bandpass filter (0.008–0.09 Hz) was applied to the residual BOLD signal [91]. The effective degrees of freedom after denoising ranged from 138.5 to 183.1 (mean = 179.7) across the participants [92].

#### 2.3.3. Functional Connectivity Analyses

Seed-based connectivity (SBC) maps and region of interest (ROI-to-ROI) connectivity matrices were computed to characterize functional connectivity patterns. These analyses were conducted using 17 HPC-ICA networks [76] and regions defined by the Harvard Center for Morphometric Analysis [93]. Functional connectivity strength was quantified using Fisher-transformed bivariate correlation coefficients derived from a weighted General Linear Model (GLM) [94]. Connectivity estimates were computed separately for each seed-target pair by modeling the association between their respective BOLD signal time series. Statistical analyses were performed at the group level using GLM [94]. A separate GLM was estimated for each voxel, with first-level connectivity measures at the corresponding voxel serving as the dependent variables. Each subject contributed an independent sample at each time point (i.e., before and after cognitive training).

Seed-based functional connectivity analyses were performed using the CONN toolbox [76], version 22.v2407. Seeds were defined a priori within five intrinsic connectivity networks of interest: default mode network (DMN), dorsal attention network (DAN), ventral attention network (VAN), frontoparietal network (FPN), and salience network (SN). Specifically, the posterior cingulate cortex (PCC) and medial prefrontal cortex (MPFC) were selected as seeds for the DMN; the intraparietal sulcus (IPS) and frontal eye fields (FEF) for the DAN; the superior temporal gyrus (STG), inferior frontal gyrus (IFG), and medial frontal gyrus (MFG) for the VAN; the left prefrontal cortex (lPFC), superior frontal gyrus (SFG), and medial temporal gyrus (MTG) for the FPN; and the anterior cingulate cortex (ACC) and insula for the SN. These regions were defined using the Harvard-Oxford atlas (RRID:SCR_001476; Harvard Center for Morphometric Analysis [93]).

Seed-to-voxel connectivity maps were generated by computing Fisher-transformed bivariate correlation coefficients between the BOLD time series of each seed region and all other voxels in the brain using a weighted general linear model [94]. Connectivity estimates were computed for each participant at two time points (pre- and post-intervention) and entered into the second level, within-subject analyses. Group-level effects were modeled using random-effects general linear models, with subject-level contrast maps (post > pre) serving as inputs.

Statistical inference was conducted using multivariate parametric statistics implemented in CONN, with voxel-level significance thresholds set at *p* < 0.001 (uncorrected) and cluster-level significance assessed using family-wise error (FWE) correction at *p* < 0.05, based on the Gaussian Random Field theory.

## 3. Results

### 3.1. Behavioral Results

The participants’ pre- and post-intervention scores are reported in Table 2. A significant improvement in episodic memory was observed following the intervention, as measured by the Rey Auditory Verbal Learning Test (RAVLT) (BH-corrected *p* = 0.048). Although increases were also found in the overall cognitive performance (ACE-R total score; *p* = 0.016) and semantic association abilities (Camel and Cactus Test; *p* = 0.057), these effects did not remain significant after adjusting for multiple comparisons.

### 3.2. Functional Connectivity Results

#### 3.2.1. ROI-to-ROI Connectivity

**Figure 1:**
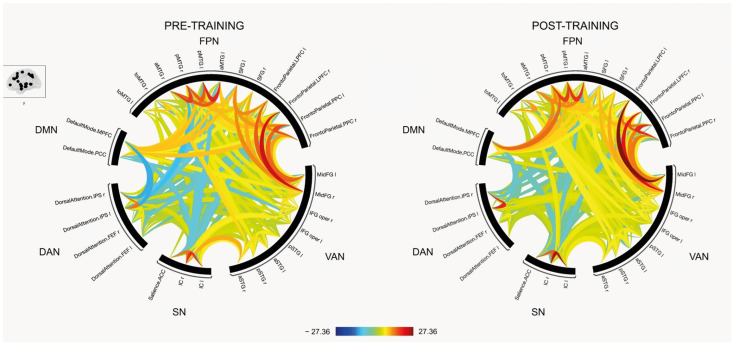
Resting state connectivity between Regions-of-interest (ROIs) pre- and post-training. The colors corresponds to t-values, with red indicating increased connectivity and blue indicating decreased connectivity (see color bar at the bottom). DMN, default mode network; FPN = Frontoparietal network; VAN = Ventral attentional network; SN = Salience network; DAN = Dorsal attentional network.

No significant differences were observed in the ROI-to-ROI connectivity analysis between pre- and post-interventions. However, a visual inspection of the connectivity patterns suggests potential network-level changes, as illustrated in Figure 1.

#### 3.2.2. Seed-to-Voxel Connectivity

**Figure 2:**
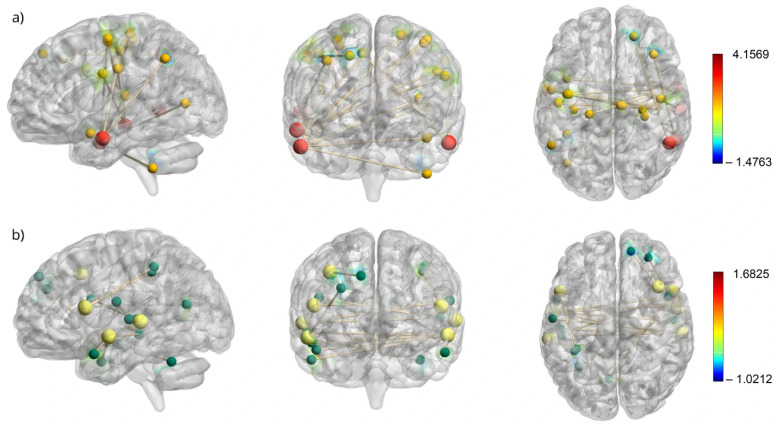
Resting state connectivity maps of FPN seeds and VAN seeds and their corresponding activated voxels. (**a**) Resting-state connectivity map for the frontoparietal network (FPN) with seed regions shown as larger red nodes and their corresponding activated voxels as smaller yellow nodes. (**b**) Resting-state connectivity map for the ventral attention network (VAN) with seed regions shown as larger yellow nodes and their corresponding activated voxels as smaller green nodes. The colorbars corresponds to t-values, with red indicating increased connectivity and blue indicating decreased connectivity.

The areas of differential changes in resting-state functional connectivity are reported in detail in Table 3. Figure 2 provides a visual representation of the seed locations and spatial extent of connectivity changes within the FPN and VAN, which exhibited the most prominent changes following the intervention program. The results for each network are summarized separately in the following section.

#### 3.2.3. Default Mode Network (DMN)

A significant decrease in functional connectivity was observed between the medial prefrontal cortex (mPFC) and right superior lateral occipital cortex. No significant changes were detected for the posterior cingulate cortex (PCC).

#### 3.2.4. The Dorsal Attention Network (DAN)

No significant changes in connectivity were found across any of the DAN seed regions.

#### 3.2.5. The Ventral Attention Network (VAN)

The inferior frontal gyrus (IFG) exhibited increased connectivity with the precuneus and superior parietal lobule (SPL). The middle frontal gyrus (MFG) showed decreased connectivity with the frontal pole (FP). The superior temporal gyrus (STG) demonstrates increased and decreased connectivity in various regions, including the temporal pole (TP), middle temporal gyrus (MTG), and cerebellar areas.

#### 3.2.6. Frontoparietal Network (FPN)

The right PCC showed decreased connectivity with the frontal pole. The MTG regions exhibited both increased and decreased connectivity with the motor and sensory cortices, as well as with the insular cortex (IC) and cerebellum.

#### 3.2.7. Salience Network (SN)

The left insular cortex (IC) showed increased connectivity with the right anterior parahippocampal gyrus and the amygdala. No significant changes were observed in the right IC or anterior cingulate gyrus.

## 4. Discussion

This study investigated the effects of a language-based cognitive intervention on brain functional connectivity in a group of older native Brazilian Portuguese-speaking women with medium to high socioeconomic status. At the behavioral level, a significant improvement in verbal episodic memory was observed, suggesting that the intervention may have contributed to enhancing the participants’ verbal memory abilities, potentially through improved encoding, storage, or retrieval processes. As hypothesized, these gains were accompanied by increased functional connectivity within the FPN, SN, and VAN networks associated with cognitive control, attentional reorientation, and salience detection. In contrast, decreased connectivity was observed within the DMN, a finding that diverges from previous cognitive training studies in older adults where increased DMN connectivity has sometimes been interpreted as compensatory.

### 4.1.  Cognitive Gains Post-Intervention

The intervention focusing on linguistic activities had a more pronounced impact on episodic memory among all the cognitive functions assessed in the pre- versus post-intervention comparison. This finding aligns with our hypotheses. In accordance with a previous study from our group, delivered online during the COVID-19 pandemic to a group of participants with similar SES, schooling, and RWH profiles, significant differences were found in episodic memory following the online intervention [25]. Considering that episodic memory decline is one of the most prominent signs of cognitive impairment in dementia [95], preventive interventions such as those reported in this study seem to be valuable for mitigating the individual, social, and governmental burdens of the increase in dementia worldwide.

In addition to the observed gains, the present results suggest that linguistic cognitive training can promote improvements across various cognitive constructs, even in individuals without established cognitive impairments or subjective memory complaints. More specifically, it was observed that even among participants with medium to high levels of schooling, there remains room for the enhancement of cognitive abilities such as memory, attention, language, and executive functions. These findings reinforce the relevance of cognitive training strategies not only in rehabilitation contexts but also as preventive and health-promoting approaches, contributing to the expansion of cognitive reserve and supporting the maintenance of intellectual functioning throughout aging. Furthermore, linguistic interventions have been shown to effectively enhance cognitive functions, such as memory and attention, in healthy populations [25] as well as in individuals with mild cognitive impairment (MCI) and Alzheimer’s disease (AD) [24]. This shows the richness and complexity of language training, since language processing mobilizes several cognitive constructs to perform language computations at both the comprehension and production levels [31]. This finding has important implications for further research on the intertwined relation between language, cognition, and the brain, as well as for schooling and clinical settings.

A systematic review by Basak et al. [96] concluded that both single-domain and multi-domain cognitive training can produce near- and far-transfer effects, with stronger evidence supporting the benefits of multi-domain approaches. However, among the included studies, none had provided specifically language-based training, which engages executive functions and relies on crystallized knowledge, while being closely tied to daily activities. Compared to episodic memory training, language-based training targets higher-order cognitive processes without relying on free or cued delayed recall tasks, which can be particularly challenging for older adults. This approach may improve adherence and facilitate greater transfer to everyday activities due to its higher ecological validity. Our findings highlight the need for further controlled studies on language-based training, focusing on adherence, enjoyability, and transfer effects compared to other training modalities.

Although overall cognitive performance (ACE-R) and semantic association ability (CCT) improved, these results did not remain statistically significant after adjustments for multiple comparisons. These trends should be considered and further explored in future interventions with longer durations and/or greater intensities and larger groups. Moreover, the absence of a significant reduction in subjective memory complaints may reflect the frequently observed dissociation between subjective perception and objective performance in cognitive assessment.

### 4.2. Functional Connectivity Changes in Response to Cognitive Training

Consistent with previous research on general cognitive training [47], the present study found that FPN showed predominantly increased functional connectivity following language-based intervention. The FPN is frequently emphasized in cognitive training literature because of its role as a higher-order cognitive hub, supporting executive control, cognitive flexibility, semantic regulation, and working memory [33,34]. In the present study, the observed increase in FPN connectivity was accompanied by significant improvements in verbal episodic memory performance. This convergence aligns with prior studies linking the FPN to successful episodic memory retrieval [97], and suggests that enhanced connectivity within this network may contribute to the observed cognitive benefits. In a 24-month longitudinal study, Hsu et al. [98] observed that older adults who maintained their gait speed exhibited greater functional connectivity within the frontoparietal network (FPN), specifically between the ventral visual cortex and supramarginal gyrus. This enhanced connectivity was positively correlated with performance on verbal episodic memory tasks, suggesting a functional link between preserved network integrity and memory outcomes during aging. Together with the current findings, these results reinforce the notion that targeted cognitive training can effectively modulate large-scale brain networks to support domain-specific cognitive functions.

Building on this broader perspective, we now turn to the specific connectivity changes within the FPN observed following linguistic-based cognitive training, although most language-specific behavioral measures did not show significant improvement, in addition to verbal episodic memory, which, while verbal, is not uniquely linguistic [31]. The primary connectivity changes within the FPN were driven by seeds in the right MTG. The MTG is a well-established semantic hub [99], that plays a central role in language comprehension and conceptual integration [100]. The observed increase in connectivity between the right MTG and precentral gyrus is particularly noteworthy given the linguistic focus of the intervention. While the phonological loop is typically supported by left-lateralized regions, such as Broca’s area and the left precentral gyrus [101], aging and cognitive training have been associated with more bilateral or compensatory recruitment of frontotemporal networks [102]. In this context, the right MTG may contribute to verbal encoding and rehearsal through enhanced interactions with motor-planning regions, especially under increased cognitive demands [103,104]. In addition to phonological processing, the precentral gyrus has been implicated in higher-order semantic operations. We hypothesized that the observed enhancement in FPN connectivity may support improvements in working memory by facilitating more efficient encoding processes. Working memory performance showed only a non-significant trend toward improvement (*p* = 0.076). The neural findings are consistent with prior work indicating that semantic control and integration can recruit bilateral middle temporal regions, particularly under conditions of greater task complexity (e.g., [105,106]). Although these studies did not examine cognitive training, they suggest that increased semantic demands can be associated with broader bilateral engagement of the semantic network. The bilateral recruitment may also represent an age-related shift toward more bilateral language processing, consistent with compensatory mechanisms described in older adults [102,107].

In addition to the changes observed within the FPN, increased connectivity was also identified in the ventral attentional network (VAN), consistent with its proposed role in language processing and cognitive control. Several studies have highlighted the role of VAN in language processing. For instance, Freedman et al. [40] demonstrated that the ability to reorient attention to salient stimuli correlates with reading fluency, suggesting that VAN may facilitate shifts in attention to internal processes during the retrieval of meaning from written stimuli. This attentional flexibility is also critical for efficiently transitioning from one word to the next, thereby supporting reading speed and comprehension. In our study, the most significant increases in VAN connectivity were observed with seeds located in the left anterior STG and posterior STG bilaterally. Notably, these seeds showed increased connectivity with the temporal regions in the contralateral hemisphere, suggesting enhanced interhemispheric coordination. Given the established role of the STG in language processing (e.g., [108,109]), these findings may reflect a strengthened capacity to dynamically reorient attention during linguistic tasks, particularly those involving semantic access and word-to-word transitions. As with the frontoparietal network (FPN), this cross-hemispheric engagement of the VAN aligns with evidence that bilateral recruitment supports compensatory processing during aging, as proposed by Cabeza et al., [102].

The salience network (SN) also exhibited enhanced connectivity following the intervention, which is consistent with Cao et al. [46] reported multi-domain cognitive training. This network plays a critical role in detecting, monitoring, and filtering salient stimuli to guide attention and cognitive control toward the most relevant information [110]. Notably, key regions of the SN, such as the anterior insula, have also been implicated in language functions including articulation, word retrieval, and phonological discrimination, suggesting a broader role in supporting language-related cognitive processes [42]. More specifically, increased connectivity was observed between the left insular cortex (core SN hub) and right parahippocampal gyrus and amygdala. While previous studies have more commonly reported SN-related increases in the right anterior insula [43,46], our findings suggest that left-lateralized SN engagement may also play a role in modulating memory-related processes, particularly in language-based cognitive training. This pattern may reflect an improved coordination between salience detection and memory-related regions, potentially enhancing the ability to filter out irrelevant stimuli and prioritize emotionally or contextually significant information during encoding. The involvement of the parahippocampal gyrus and amygdala further suggests enhanced integration of attentional and affective cues, which is especially relevant in aging populations, where attentional control often declines.

In contrast to previous studies [46,47,48] the present findings revealed a decrease in connectivity within the default mode network (DMN), specifically between the medial prefrontal cortex and the lateral occipital cortex, following language-based cognitive training. Given the linguistic and externally oriented nature of the intervention, this reduction in DMN connectivity may reflect an adaptive disengagement from internally directed processes, such as mind-wandering and self-referential thought. Such disengagement could facilitate a more efficient recruitment of task-positive networks involved in attention and goal-directed behavior. This interpretation aligns with prior research indicating that the suppression of DMN activity is associated with improved performance during externally focused cognitive tasks [37,111,112]. Moreover, increased anterior DMN connectivity has been observed in the early stages of Alzheimer’s disease, often interpreted as a loss of network specificity or compensatory overactivation [113]. In this context, the observed decrease in connectivity may represent beneficial modulation, potentially contributing to more efficient neural processing and cognitive resilience.

Unsurprisingly, no significant changes in connectivity were observed in the dorsal attentional network (DAN), which is consistent with previous studies involving cognitively healthy older adults [49,50]. The DAN is primarily engaged during top-down, goal-directed visuospatial attention tasks such as visual search, spatial orienting, and attentional tracking, which are not directly targeted by the present intervention. As such, the absence of a change may reflect the specificity of other networks. Greenwood and Parasuraman [51] further suggested that the lack of DAN modulation in older adults following cognitive interventions may stem from high levels of schooling or cognitive reserve, which could buffer training-related neural changes in this network. Alternatively, it may reflect the network’s limited involvement in far-transfer mechanisms, particularly when trained tasks do not overlap with the attentional demands typically mediated by the DAN.

Although the results of the seed-to-voxel analysis are promising and suggest localized changes in functional connectivity following the intervention, they should be interpreted with caution. Seed-to-voxel methods are inherently more exploratory and sensitive to spatial focal effects, which may not be captured by the more constrained ROI-to-ROI approach. The lack of significant findings in the ROI-to-ROI analysis may reflect the limited statistical power, as only 17 participants were included in the connectivity analysis. Given the small sample size, it is possible that subtle but meaningful effects were detected at the voxel level but were not robust enough to emerge when averaged across broader anatomical ROIs. These findings highlight the need for replication in larger samples and suggest that seed-to-voxel results can offer valuable preliminary insights into training-induced brain plasticity. Because only women were included, the findings cannot be generalized to men. In addition, the sample consisted of highly educated participants, which is not representative of the broader Brazilian population. The absence of an active control group further limits causal inferences regarding the intervention’s specific effects. Future studies should therefore include larger and more balanced samples of both sexes, a wider range of educational backgrounds, and appropriate control groups.

Another limitation of the present study should be acknowledged. First, part of the cognitive training program consisted of home-based individual activities designed to last approximately 30–40 min per day over four days each week, in addition to weekly face-to-face sessions. While these home activities likely contributed to maintaining participants’ engagement and participants reported adhering to the schedule as instructed, researchers had limited ability to monitor or control this component of the intervention. Finally, the relatively brief duration of the intervention and the absence of a follow-up assessment prevent us from determining the durability of the observed effects. The long-term impact of cognitive training programs, both in terms of behavioral outcomes and brain connectivity changes, remains unknown and should be explored in future studies.

## 5. Conclusions

This study provides compelling evidence that a language-based cognitive training intervention can yield measurable cognitive and neurofunctional benefits in cognitively healthy older adults from middle-to-high socioeconomic backgrounds. These findings underscore the capacity for cognitive enhancement, even among individuals with higher schooling levels, highlighting the relevance of preventive strategies beyond traditional clinical rehabilitation.

Importantly, the results support the hypothesis that targeted language-based cognitive training can strengthen neural and cognitive mechanisms, particularly those involved in verbal episodic memory, thereby contributing to the enhancement of cognitive reserve. These results have significant implications for aging populations, suggesting that such interventions may serve as vital components in strategies aimed at promoting healthy cognitive aging.

Our findings open promising avenues for future research on scalable, non-pharmacological interventions. Further investigation of their long-term efficacy and broader applicability could provide essential tools for addressing the increasing societal burden of age-related cognitive decline and dementia.

## Figures and Tables

**Table 1:** Sociodemographic Profile and Mental Health, Cognitive, and Functional Assessment Results.

Participants n = 20			
Variable	Mean	SD	Range
Sociodemographic data			
Age (years)	69.30	4.64	63–77
Schooling (years)	14.85	3.25	9–20
Socioeconomic status †	28.25	8.46	15–49
Reading and writing habits §	48.60	16.16	14–74
Emotional, functional and cognitive assessment			
Geriatric Depression Scale	2.15	2.46	0–9
Geriatric Anxiety Inventory	6.40	5.79	0–17
Functional Activities Questionnaire	53.80	2.53	45–55
Addenbrooke’s Cognitive Examination–Revised	89.63	5.96	82–98

Note: † Socioeconomic status (SES) was assessed using Critério Brasil 2024 [59], scores were categorized into six socioeconomic groups, from highest to lowest: A (45–100 points), B1 (38–44 points), B2 (29–37 points), C1 (23–28 points), C2 (17–22 points), and DE (0–16 points). § Reading and writing habits (RWH) were assessed using the questionnaire developed by Pacheco [60].

**Table 2:** Pre- to Post-Intervention Changes in Behavioral Performance Following Language-Based Cognitive Training.

	Pre		Post				
Test	Mean	SD	Mean	SD	*W*	*p*	BH-Corrected *p*
RAVLT	43.9	8.73	49.35	8.56	169.0	**0.003**	**0.036**
ACE-R	89.95	5.97	92.20	5.24	131.0	0.010	0.060
Camel and Cactus Test	31.0	2.96	32.25	2.77	140.5	0.069	0.218
Digit Span-inverse	2.80	0.89	3.30	0.92	61.5	0.076	0.218
Phonemic verbal fluency (/p/)	14.07	4.64	16.25	4.85	150.5	0.091	0.218
Stroop word	1.27	0.16	1.22	0.23	64.0	0.133	0.245
Stroop color-word	2.14	0.57	1.97	0.51	65.0	0.143	0.245
Subjective memory complaint	32.67	10.4	32.5	8.49	64.0	0.569	0.787
Semantic verbal fluency (animals)	19.05	5.04	20.0	4.6	84.5	0.721	0.778
Digit Span-direct	4.45	1.1	4.55	0.94	26.0	0.713	0.787
Color trails test	1.23	0.57	1.12	0.48	91.0	0.622	0.787
Naming (BALE)	55.95	2.68	56.15	3.07	49.0	0.833	0.934

Notes: Significant results are shown in bold (*p* < 0.05). SD = standard deviation; *W* = Wilcoxon rank-sum test; *p* = *p*-value (statistical significance); BH-corrected *p* = *p*-value adjusted using the Benjamini–Hochberg procedure for multiple comparisons; RAVLT = Rey Auditory Verbal Learning Test [67]; ACE-R = Addenbrooke’s Cognitive Examination-Revised [64,65]; BALE = Battery for Language Assessment in Aging (BALE) [72].

**Table 3:** Seed-to-Voxel Functional Connectivity Changes Following Language-Based Cognitive Training.

Seed Region	Connected Regions	Hemisphere	MNI Coordinates x, y, z	Cluster	*t*	k	p-FWE
Default Mode Network (DMN)							
Posterior cingulate cortex	No significant results						
Medial prefrontal cortex	Superior lateral occipital cortex	Right	+44, −64, +42	1	−5.50	320	<0.001
Dorsal Attention Network (DAN)							
Left intraparietal sulcus	No significant results						
Right intraparietal sulcus	No significant results						
Left frontal eye field	No significant results						
Right frontal eye field	No significant results						
Ventral Attention Network (VAN)							
Left IFG *pars opercularis*	Precuneus	Left	−6, −66, +18	2	6.67	193	<0.001
Right IFG *pars opercularis*	Superior parietal lobule	Left	−38, −40, +48	3	6.22	182	<0.001
	Postcentral gyrus	Left	−36, −38, +44	3	6.22	182	<0.001
Left middle frontal gyrus	No significant results						
Right middle frontal gyrus	Frontal pole	Right	+14, +52, +40	4	−6.23	99	0.020
Left anterior STG	Temporal pole,	Right	+50, +10, −24	5	6.63	305	<0.001
	Anterior middle temporal gyrus	Right	+54, +2, −28				
	Temporal pole	Left	−54, +8, −22	6	6.37	112	0.011
	Posterior middle temporal gyrus	Right	+58, −28, −6	7	6.04	90	0.033
	Posterior middle temporal gyrus	Right	+54, −12, −14	8	5.87	86	0.041
Right anterior STG	Frontal pole	Right	+30, +46, +30	9	−7.50	238	<0.001
	Cerebellum 6	Left	−34, −54, −28	10	−7.05	85	0.039
Left posterior STG	Temporal pole	Right	+48, +10, −22	11	6.56	148	0.002
Right posterior STG	Planum temporale,	Left	−50, −26, +6	12	5.85	109	0.011
	Heschl’s gyrus	Left	−50, −20, +6				
	Postcentral gyrus	Left	−58, −10, +20	13	5.24	100	0.018
Frontoparietal Network (FPN)							
Left posterior parietal cortex	No significant results						
Right posterior parietal cortex	Frontal pole	Right	+20, +44, +46	14	−5.67	108	0.010
Left posterior MTG	No significant results						
Right posterior MTG	Precentral gyrus,	Left	−54, −4, +30	15	6.96	430	<0.001
	Postcentral gyrus	Left	−48, −16, +34				
	Precentral gyrus	Left	−40, −8, +58	16	6.15	173	<0.001
	Precentral gyrus	Right	+8, −18, +48	17	5.33	103	0.014
	Precentral gyrus	Right	+44, −12, +48	18	5.05	83	0.041
Left temporo-occipital MTG	No significant results						
Right temporo-occipital MTG	Medial frontal gyrus	Right	+38, +28, +42	19	−6.96	146	0.003
Left anterior MTG	Angular gyrus	Left	−54, −54, +42	20	−5.98	87	0.023
Left anterior MTG	Precentral gyrus	Right	+10, −18, +48	21	6.80	82	0.031
Right anterior MTG	Precentral	Right	+28, −22, +54	22	5.49	683	<0.001
	Postcentral gyrus		+30, −28, +58				
	Cerebellum 8	Left	−40, −44, −46	23	−9.16	217	<0.001
	Precentral gyrus	Left	−20, −26, +62	24	5.20	208	<0.001
	Insular cortex	Left	−34, −22, +12	25	6.21	99	0.015
	Temporal pole	Left	−40, +6, −18	26	5.84	94	0.019
	Inferior lateral occipital cortex	Left	−40, −70, +6	27	5.11	80	0.042
	Precentral gyrus	Left	−38, −8, +56	28	5.57	79	0.044
Left superior frontal gyrus	No significant results						
Right superior frontal gyrus	No significant results						
Left lateral prefrontal cortex	No significant results						
Right lateral prefrontal cortex	No significant results						
Salience Network (SN)							
Anterior cingulate cortex	No significant results						
Left insular cortex	Anterior parahippocampal gyrus	Right	+18, −6, −26	29	7.68	114	0.006
	Amygdala	Right	+18, −6, −20				
Right insular cortex	No significant results						

Note: IFG: inferior frontal gyrus, STG: superior temporal gyrus, MTG: Middle temporal gyrus, DMN: default mode network, VAN: ventral attention network, FPN: frontoparietal network, MNI: Montreal Neurological Institute.

## Data Availability

The data presented in this study are available on request from the corresponding author. The participants of this study did not give written consent for their data to be shared publicly, so due to the sensitive nature of the research supporting data is not available.

## Abbreviations

The following abbreviations are used in this manuscript:

ACE-R	Addenbrooke’s Cognitive Examination–Revised
ACTIVE	Advanced Training for Independent and Vital Elderly (study)
AD	Alzheimer’s disease
BALE	Battery for Language Assessment in Aging
BH	Benjamini–Hochberg (correction)
BOLD	Blood-Oxygen-Level-Dependent
CCT	Camel and Cactus Test
CEN	Central executive network
CSF	Cerebrospinal Fluid
CTT	Colors Trail Test
DAN	Dorsal attention network
DMN	Default mode network
ECN	Executive control network
FAQ	Functional Activities Questionnaire
FOV	Field of view
FP	Frontal pole
FPN	Frontoparietal network
fMRI	Functional magnetic resonance imaging
FWE	Family-wise error
GAI	Geriatric Anxiety Inventory
GDS	Geriatric Depression Scale
GLM	General Linear Model
GRE-EPI	Gradient-recalled echo planar imaging (sequence)
IC	Insular cortex
IADL	Instrumental activities of daily living
IFG	Inferior frontal gyrus
MFG	Middle frontal gyrus
MNI	Montreal Neurological Institute
MMSE	Mini-Mental State Examination
MP-RAGE	Magnetization-prepared rapid gradient echo (sequence)
mPFC	Medial prefrontal cortex
MTG	Middle temporal gyrus
PCC	Posterior cingulate cortex
RAVLT	Rey Auditory Verbal Learning Test
ROI	Region of interest
RWH	Reading and writing habits
SBC	Seed-based connectivity
SD	Standard deviation
SES	Socioeconomic status
SN	Salience network
SPL	Superior parietal lobule
STG	Superior temporal gyrus
TE	Echo time
TI	Inversion time
TP	Temporal pole
TR	Repetition time
VAN	Ventral attention network
